# Supplementary material for: Iterative taxonomic study of Pareiorhaphis hystrix (Siluriformes, Loricariidae) suggests a single, yet phenotypically variable, species in south Brazil
Source: PLoS One. 2020 Sep 3;15(9):e0237160. doi: 10.1371/journal.pone.0237160 (PMC7470336; doi:10.1371/journal.pone.0237160)
Supplement: S1 Table — Specimens used for morphological analyses are marked MORPH in the Gene column. (DOCX) [file pone.0237160.s001.docx]

**Table S1. Information of specimens used in morphological and molecular analyses of *Pareiorhaphis*.** Specimens used for morphological analyses

are marked MORPH in the column Gene.

| Species | Catalog # | Gene | Individ | Drainage | River Name | Locality (county,state) | Geographic Coordinates | GenBank Accession |
| --- | --- | --- | --- | --- | --- | --- | --- | --- |
| *P. hystrix* | MCP 53197 | *coI* | PF71 | Taquari-Antas | Upper Antas | São José dos Ausentes, RS | 28°47'04"S 49°58'57”W | MT802339 |
| *P. hystrix* | MCP 53197 | *cytb* | PF71 | Taquari-Antas | Upper Antas | São José dos Ausentes, RS | 28°47'04"S 49°58'57”W | MT809648 |
| *P. hystrix* | MCP 53197 | 16S | PF71 | Taquari-Antas | Upper Antas | São José dos Ausentes, RS | 28°47'04"S 49°58'57”W | MT799915 |
| *P. hystrix* | MCP 53197 | 12S | PF71 | Taquari-Antas | Upper Antas | São José dos Ausentes, RS | 28°47'04"S 49°58'57”W | MT803126 |
| *P. hystrix* | MCP 53197 | *coI* | PF72 | Taquari-Antas | Upper Antas | São José dos Ausentes, RS | 28°47'04"S 49°58'57”W | MT802340 |
| *P. hystrix* | MCP 53197 | *cytb* | PF72 | Taquari-Antas | Upper Antas | São José dos Ausentes, RS | 28°47'04"S 49°58'57”W | MT809649 |
| *P. hystrix* | MCP 53197 | 16S | PF72 | Taquari-Antas | Upper Antas | São José dos Ausentes, RS | 28°47'04"S 49°58'57”W | MT799916 |
| *P. hystrix* | MCP 53197 | 12S | PF72 | Taquari-Antas | Upper Antas | São José dos Ausentes, RS | 28°47'04"S 49°58'57”W | MT803127 |
| *P. hystrix* | MCP 35069 | MORPH | PH160 | Taquari-Antas | Upper Antas | São José dos Ausentes, RS | 28°47'03"S 49°58'57”W | - |
| *P. hystrix* | MCP 35069 | MORPH | PH163 | Taquari-Antas | Upper Antas | São José dos Ausentes, RS | 28°47'03"S 49°58'57”W | - |
| *P. hystrix* | UFRGS 8910 | MORPH | PH173 | Taquari-Antas | Upper Antas | São José dos Ausentes, RS | 28°47'01"S 49°58'57”W | - |
| *P. hystrix* | UFRGS 8910 | MORPH | PH174 | Taquari-Antas | Upper Antas | São José dos Ausentes, RS | 28°47'01"S 49°58'57”W | - |
| *P. hystrix* | UFRGS 8910 | MORPH | PH174b | Taquari-Antas | Upper Antas | São José dos Ausentes, RS | 28°47'01"S 49°58'57”W | - |
| *P. hystrix* | UFRGS 8911 | MORPH | PH170 | Taquari-Antas | Upper Antas | São José dos Ausentes, RS | 28°47'01"S 49°58'56”W | - |
| *P. hystrix* | UFRGS 8911 | MORPH | PH171 | Taquari-Antas | Upper Antas | São José dos Ausentes, RS | 28°47'01"S 49°58'56”W | - |
| *P. hystrix* | MCP 25666 | MORPH | PH154 | Taquari-Antas | Upper Antas | São José dos Ausentes, RS | ≈ 28°47'S 49°59'W | - |
| *P. hystrix* | MCP 25666 | MORPH | PH155 | Taquari-Antas | Upper Antas | São José dos Ausentes, RS | ≈ 28°47'S 49°59'W | - |
| *P. hystrix* | MCP 25668 | MORPH | PH156 | Taquari-Antas | Upper Antas | São José dos Ausentes, RS | ≈ 28°47'S 49°59'W | - |
| *P. hystrix* | MCP 25668 | MORPH | PH157 | Taquari-Antas | Upper Antas | São José dos Ausentes, RS | ≈ 28°47'S 49°59'W | - |
| *P. hystrix* | MCP 25668 | MORPH | PH162 | Taquari-Antas | Upper Antas | São José dos Ausentes, RS | ≈ 28°47'S 49°59'W | - |
| *P. hystrix* | MCP 25668 | MORPH | PH165 | Taquari-Antas | Upper Antas | São José dos Ausentes, RS | ≈ 28°47'S 49°59'W | - |
| *P. hystrix* | MCP 25668 | MORPH | PH166 | Taquari-Antas | Upper Antas | São José dos Ausentes, RS | ≈ 28°47'S 49°59'W | - |
| *P. hystrix* | MCP 35068 | MORPH | PH153 | Taquari-Antas | Upper Antas | São José dos Ausentes, RS | 28°48'36"S 49°59'37”W | - |
| *P. hystrix* | MCP 35068 | MORPH | PH164 | Taquari-Antas | Upper Antas | São José dos Ausentes, RS | 28°48'36"S 49°59'37”W | - |
| *P. hystrix* | UFRGS 21850 | MORPH | PH172 | Taquari-Antas | Upper Antas | São José dos Ausentes, RS | 28°52'09"S 50°01'14”W | - |
| *P. hystrix* | UNISINOS 53 | *coI* | PF1 | Taquari-Antas | Upper Antas | Cambará do Sul, RS | 29°09'25''S 50°03'06''W | MT802316 |
| *P. hystrix* | UNISINOS 53 | *cytb* | PF1 | Taquari-Antas | Upper Antas | Cambará do Sul, RS | 29°09'25''S 50°03'06''W | MT809618 |
| *P. hystrix* | UNISINOS 53 | 16S | PF1 | Taquari-Antas | Upper Antas | Cambará do Sul, RS | 29°09'25''S 50°03'06''W | MT799885 |
| *P. hystrix* | UNISINOS 53 | 12S | PF1 | Taquari-Antas | Upper Antas | Cambará do Sul, RS | 29°09'25''S 50°03'06''W | MT803096 |
| *P. hystrix* | UNISINOS 97 | *coI* | PF2 | Taquari-Antas | Upper Antas | Cambará do Sul, RS | 29°09'25''S 50°03'06''W | MT802317 |
| *P. hystrix* | UNISINOS 97 | *cytb* | PF2 | Taquari-Antas | Upper Antas | Cambará do Sul, RS | 29°09'25''S 50°03'06''W | MT809619 |
| *P. hystrix* | UNISINOS 97 | 16S | PF2 | Taquari-Antas | Upper Antas | Cambará do Sul, RS | 29°09'25''S 50°03'06''W | MT799886 |
| *P. hystrix* | UNISINOS 97 | 12S | PF2 | Taquari-Antas | Upper Antas | Cambará do Sul, RS | 29°09'25''S 50°03'06''W | MT803097 |
| *P. hystrix* | MCP 50156 | MORPH | PH158 | Taquari-Antas | Upper Antas | Cambará do Sul, RS | 28°52'12"S 50°01'13”W | - |
| *P. hystrix* | MCP 50156 | MORPH | PH159 | Taquari-Antas | Upper Antas | Cambará do Sul, RS | 28°52'12"S 50°01'13”W | - |
| *P. hystrix* | MCP 50156 | MORPH | PH161 | Taquari-Antas | Upper Antas | Cambará do Sul, RS | 28°52'12"S 50°01'13”W | - |
| *P. hystrix* | UNISINOS 2368 | MORPH | PH168 | Taquari-Antas | Upper Antas | Cambará do Sul, RS | 29°06'14''S 50°07'40”W | - |
| *P. hystrix* | MCP 53186 | MORPH | PH175 | Taquari-Antas | Upper Antas | Cambará do Sul, RS | 29°10'46"S 50°08'13”W | - |
| *P. hystrix* | MCP 53186 | MORPH | PH176 | Taquari-Antas | Upper Antas | Cambará do Sul, RS | 29°10'46"S 50°08'13”W | - |
| *P. hystrix* | MCP 53186 | MORPH | PH177 | Taquari-Antas | Upper Antas | Cambará do Sul, RS | 29°10'46"S 50°08'13”W | - |
| *P. hystrix* | MCP 53186 | *coI* | PF70 | Taquari-Antas | Upper Antas | Cambará do Sul, RS | 29°10'46"S 50°08'13”W | MT802338 |
| *P. hystrix* | MCP 53186 | *cytb* | PF70 | Taquari-Antas | Upper Antas | Cambará do Sul, RS | 29°10'46"S 50°08'13”W | MT809647 |
| *P. hystrix* | MCP 53186 | 16S | PF70 | Taquari-Antas | Upper Antas | Cambará do Sul, RS | 29°10'46"S 50°08'13”W | MT799914 |
| *P. hystrix* | MCP 53186 | 12S | PF70 | Taquari-Antas | Upper Antas | Cambará do Sul, RS | 29°10'46"S 50°08'13”W | MT803125 |
| *P. hystrix* | UNISINOS 842 | MORPH | PH167 | Taquari-Antas | Upper Antas | Cambará do Sul, RS | 29°10'45"S 50°08'13"W | - |
| *P. hystrix* | UNISINOS 2579 | MORPH | PH169 | Taquari-Antas | Upper Antas | Cambará do Sul, RS | 29°06'04"S 50°06'50”W | - |
| *P. hystrix* | MCP 53190 | MORPH | PH18 | Taquari-Antas | Upper Antas | Tainhas, RS | 29°12'34"S 50°14'19”W | - |
| *P. hystrix* | MCP 53190 | MORPH | PH19 | Taquari-Antas | Upper Antas | Tainhas, RS | 29°12'34"S 50°14'19”W | - |
| *P. hystrix* | MCP 53190 | MORPH | PH20 | Taquari-Antas | Upper Antas | Tainhas, RS | 29°12'34"S 50°14'19”W | - |
| *P. hystrix* | MCP 50093 | *coI* | PF92 | Taquari-Antas | Upper Antas | Tainhas, RS | 29°12'32"S 50°14'17”W | MT802352 |
| *P. hystrix* | MCP 50093 | *cytb* | PF92 | Taquari-Antas | Upper Antas | Tainhas, RS | 29°12'32"S 50°14'17”W | MT809662 |
| *P. hystrix* | MCP 50093 | 16S | PF92 | Taquari-Antas | Upper Antas | Tainhas, RS | 29°12'32"S 50°14'17”W | MT799929 |
| *P. hystrix* | MCP 50093 | 12S | PF92 | Taquari-Antas | Upper Antas | Tainhas, RS | 29°12'32"S 50°14'17”W | MT803140 |
| *P. hystrix* | MCP 41308 | MORPH | PH6 | Taquari-Antas | Upper Antas | Tainhas, RS | 29°16'43"S 50°14'41”W | - |
| *P. hystrix* | MCP 48714 | *coI* | PF91 | Taquari-Antas | Upper Antas | Tainhas, RS | 29°16'56"S 50°22'60”W | MT802351 |
| *P. hystrix* | MCP 48714 | *cytb* | PF91 | Taquari-Antas | Upper Antas | Tainhas, RS | 29°16'56"S 50°22'60”W | MT809661 |
| *P. hystrix* | MCP 48714 | 16S | PF91 | Taquari-Antas | Upper Antas | Tainhas, RS | 29°16'56"S 50°22'60”W | MT799928 |
| *P. hystrix* | MCP 48714 | 12S | PF91 | Taquari-Antas | Upper Antas | Tainhas, RS | 29°16'56"S 50°22'60”W | MT803139 |
| *P. hystrix* | MCP 53185 | MORPH | PH21 | Taquari-Antas | Upper Antas | Tainhas, RS | 29°15'03"S 50°16'42”W | - |
| *P. hystrix* | MCP 53187 | MORPH | PH16 | Taquari-Antas | Upper Antas | Tainhas, RS | 29°14'02"S 50°22'30”W | - |
| *P. hystrix* | MCP 53187 | MORPH | PH17 | Taquari-Antas | Upper Antas | Tainhas, RS | 29°14'02"S 50°22'30”W | - |
| *P. hystrix* | MCP 53187 | *coI* | PF90 | Taquari-Antas | Upper Antas | Tainhas, RS | 29°14'02"S 50°22'30”W | MT802350 |
| *P. hystrix* | MCP 53187 | *cytb* | PF90 | Taquari-Antas | Upper Antas | Tainhas, RS | 29°14'02"S 50°22'30”W | MT809660 |
| *P. hystrix* | MCP 53187 | 16S | PF90 | Taquari-Antas | Upper Antas | Tainhas, RS | 29°14'02"S 50°22'30”W | MT799927 |
| *P. hystrix* | MCP 53187 | 12S | PF90 | Taquari-Antas | Upper Antas | Tainhas, RS | 29°14'02"S 50°22'30”W | MT803138 |
| *P. hystrix* | MCP 22346 | MORPH | PH1 | Taquari-Antas | Upper Antas | Tainhas, RS | 29°13'57"S 50°22'33”W | - |
| *P. hystrix* | MCP 53200 | MORPH | PH195 | Taquari-Antas | Middle Antas | Bom Jesus, RS | 28°46'49"S 50°28'48”W | - |
| *P. hystrix* | MCP 53200 | MORPH | PH196 | Taquari-Antas | Middle Antas | Bom Jesus, RS | 28°46'49"S 50°28'48”W | - |
| *P. hystrix* | MCP 53200 | *coI* | PF79 | Taquari-Antas | Middle Antas | Bom Jesus, RS | 28°46'49"S 50°28'48”W | MT802347 |
| *P. hystrix* | MCP 53200 | *cytb* | PF79 | Taquari-Antas | Middle Antas | Bom Jesus, RS | 28°46'49"S 50°28'48”W | MT809656 |
| *P. hystrix* | MCP 53200 | 16S | PF79 | Taquari-Antas | Middle Antas | Bom Jesus, RS | 28°46'49"S 50°28'48”W | MT799923 |
| *P. hystrix* | MCP 53200 | 12S | PF79 | Taquari-Antas | Middle Antas | Bom Jesus, RS | 28°46'49"S 50°28'48”W | MT803134 |
| *P. hystrix* | MCP 22793 | MORPH | PH180 | Taquari-Antas | Middle Antas | Bom Jesus, RS | 28°46'48"S 50°28'48”W | - |
| *P. hystrix* | MCP 22793 | MORPH | PH181 | Taquari-Antas | Middle Antas | Bom Jesus, RS | 28°46'48"S 50°28'48”W | - |
| *P. hystrix* | MCP 22793 | MORPH | PH182 | Taquari-Antas | Middle Antas | Bom Jesus, RS | 28°46'48"S 50°28'48”W | - |
| *P. hystrix* | MCP 22793 | MORPH | PH183 | Taquari-Antas | Middle Antas | Bom Jesus, RS | 28°46'48"S 50°28'48”W | - |
| *P. hystrix* | MCP 53184 | MORPH | PH199 | Taquari-Antas | Middle Antas | Lageado Grande, RS | 29°02'21"S 50°34'09”W | - |
| *P. hystrix* | MCP 53184 | *coI* | PF78 | Taquari-Antas | Middle Antas | Lageado Grande, RS | 29°02'21"S 50°34'09”W | MT802346 |
| *P. hystrix* | MCP 53184 | *cytb* | PF78 | Taquari-Antas | Middle Antas | Lageado Grande, RS | 29°02'21"S 50°34'09”W | MT809655 |
| *P. hystrix* | MCP 53184 | 16S | PF78 | Taquari-Antas | Middle Antas | Lageado Grande, RS | 29°02'21"S 50°34'09”W | MT799922 |
| *P. hystrix* | MCP 53184 | 12S | PF78 | Taquari-Antas | Middle Antas | Lageado Grande, RS | 29°02'21"S 50°34'09”W | MT803133 |
| *P. hystrix* | MCP 50159 | MORPH | PH191 | Taquari-Antas | Middle Antas | Lageado Grande, RS | 29°02'21"S 50°34'08”W | - |
| *P. hystrix* | MCP 50159 | MORPH | PH192 | Taquari-Antas | Middle Antas | Lageado Grande, RS | 29°02'21"S 50°34'08”W | - |
| *P. hystrix* | MCP 50159 | MORPH | PH193 | Taquari-Antas | Middle Antas | Lageado Grande, RS | 29°02'21"S 50°34'08”W | - |
| *P. hystrix* | MCP 50159 | *coI* | PF41 | Taquari-Antas | Middle Antas | Lageado Grande, RS | 29°02'21"S 50°34'08”W | MT802329 |
| *P. hystrix* | MCP 50159 | *cytb* | PF41 | Taquari-Antas | Middle Antas | Lageado Grande, RS | 29°02'21"S 50°34'08”W | MT809634 |
| *P. hystrix* | MCP 50159 | 16S | PF41 | Taquari-Antas | Middle Antas | Lageado Grande, RS | 29°02'21"S 50°34'08”W | MT799901 |
| *P. hystrix* | MCP 50159 | 12S | PF41 | Taquari-Antas | Middle Antas | Lageado Grande, RS | 29°02'21"S 50°34'08”W | MT803112 |
| *P. hystrix* | MCP 50159 | *coI* | PF42 | Taquari-Antas | Middle Antas | Lageado Grande, RS | 29°02'21"S 50°34'08”W | MT802330 |
| *P. hystrix* | MCP 50159 | *cytb* | PF42 | Taquari-Antas | Middle Antas | Lageado Grande, RS | 29°02'21"S 50°34'08”W | MT809635 |
| *P. hystrix* | MCP 50159 | 16S | PF42 | Taquari-Antas | Middle Antas | Lageado Grande, RS | 29°02'21"S 50°34'08”W | MT799902 |
| *P. hystrix* | MCP 50159 | 12S | PF42 | Taquari-Antas | Middle Antas | Lageado Grande, RS | 29°02'21"S 50°34'08”W | MT803113 |
| *P. hystrix* | MCP 22351 | MORPH | PH190 | Taquari-Antas | Middle Antas | Lageado Grande, RS | 29°05'34"S 50°37'30”W | - |
| *P. hystrix* | MCP 48729 | MORPH | PH188 | Taquari-Antas | Middle Antas | Lageado Grande, RS | 29°05'35"S 50°37'32”W | - |
| *P. hystrix* | MCP 48729 | MORPH | PH189 | Taquari-Antas | Middle Antas | Lageado Grande, RS | 29°05'35"S 50°37'32”W | - |
| *P. hystrix* | MCP 42786 | MORPH | PH201 | Taquari-Antas | Middle Antas | Bom Jesus, RS | 28°44'02"S 50°40'42”W | - |
| *P. hystrix* | MCP 42786 | MORPH | PH202 | Taquari-Antas | Middle Antas | Bom Jesus, RS | 28°44'02"S 50°40'42”W | - |
| *P. hystrix* | MCP 42786 | MORPH | PH202B | Taquari-Antas | Middle Antas | Bom Jesus, RS | 28°44'02"S 50°40'42”W | - |
| *P. hystrix* | MCP 42786 | MORPH | PH202C | Taquari-Antas | Middle Antas | Bom Jesus, RS | 28°44'02"S 50°40'42”W | - |
| *P. hystrix* | MCP 48711 | *coI* | PF93 | Taquari-Antas | Middle Antas | Bom Jesus, RS | 28°44'24"S 50°40'43”W | MT802353 |
| *P. hystrix* | MCP 48711 | *cytb* | PF93 | Taquari-Antas | Middle Antas | Bom Jesus, RS | 28°44'24"S 50°40'43”W | MT809663 |
| *P. hystrix* | MCP 48711 | 16S | PF93 | Taquari-Antas | Middle Antas | Bom Jesus, RS | 28°44'24"S 50°40'43”W | MT799930 |
| *P. hystrix* | MCP 48711 | 12S | PF93 | Taquari-Antas | Middle Antas | Bom Jesus, RS | 28°44'24"S 50°40'43”W | MT803141 |
| *P. hystrix* | MCP 43972 | MORPH | PH185 | Taquari-Antas | Middle Antas | Bom Jesus, RS | 28°44'23"S 50°40'42"W | - |
| *P. hystrix* | MCP 43343 | MORPH | PH178 | Taquari-Antas | Middle Antas | Bom Jesus, RS | 28°44'23"S 50°40'42”W | - |
| *P. hystrix* | MCP 43343 | MORPH | PH179 | Taquari-Antas | Middle Antas | Bom Jesus, RS | 28°44'23"S 50°40'42”W | - |
| *P. hystrix* | MCP 43343 | MORPH | ´PH186 | Taquari-Antas | Middle Antas | Bom Jesus, RS | 28°44'23"S 50°40'42”W | - |
| *P. hystrix* | MCP 42792 | MORPH | PH200 | Taquari-Antas | Middle Antas | Bom Jesus, RS | 28°47'42"S 50°42'18”W | - |
| *P. hystrix* | MCP 48327 | MORPH | PH184 | Taquari-Antas | Middle Antas | São Francisco de Paula, RS | 28°48’59"S 50°46’31”W | - |
| *P. hystrix* | MCP 32405 | MORPH | PH187 | Taquari-Antas | Middle Antas | São Francisco de Paula, RS | 29°02'19"S 50°34'07”W | - |
| *P. hystrix* | MCP 43518 | MORPH | PH127 | Taquari-Antas | Middle Antas | Flores da Cunha, RS | 28°56'33"S 51°14'12”W | - |
| *P. hystrix* | MCP 22787 | MORPH | PH251 | Taquari-Antas | Prata | Muitos Capões, RS | 28°23'26"S 51°03'22"W | - |
| *P. hystrix* | MCP 22787 | MORPH | PH252 | Taquari-Antas | Prata | Muitos Capões, RS | 28°23'26"S 51°03'22"W | - |
| *P. hystrix* | MCP 22787 | MORPH | PH253 | Taquari-Antas | Prata | Muitos Capões, RS | 28°23'26"S 51°03'22"W | - |
| *P. hystrix* | MCP 22787 | MORPH | PH254 | Taquari-Antas | Prata | Muitos Capões, RS | 28°23'26"S 51°03'22"W | - |
| *P. hystrix* | MCP 22787 | MORPH | PH255 | Taquari-Antas | Prata | Muitos Capões, RS | 28°23'26"S 51°03'22"W | - |
| *P. hystrix* | MCP 22787 | MORPH | PH256 | Taquari-Antas | Prata | Muitos Capões, RS | 28°23'26"S 51°03'22"W | - |
| *P. hystrix* | MCP 22787 | MORPH | PH257 | Taquari-Antas | Prata | Muitos Capões, RS | 28°23'26"S 51°03'22"W | - |
| *P. hystrix* | MCP 35066 | MORPH | PH246 | Taquari-Antas | Prata | Muitos Capões, RS | 28°23'26"S 51°03'22”W | - |
| *P. hystrix* | MCP 49481 | MORPH | PH258 | Taquari-Antas | Prata | Muitos Capões, RS | 28°21'53"S 51°17'53”W | - |
| *P. hystrix* | MCP 49481 | *coI* | PF82 | Taquari-Antas | Prata | Muitos Capões, RS | 28°21'53"S 51°17'53”W | MT802349 |
| *P. hystrix* | MCP 49481 | *cytb* | PF82 | Taquari-Antas | Prata | Muitos Capões, RS | 28°21'53"S 51°17'53”W | MT809659 |
| *P. hystrix* | MCP 49481 | 16S | PF82 | Taquari-Antas | Prata | Muitos Capões, RS | 28°21'53"S 51°17'53”W | MT799926 |
| *P. hystrix* | MCP 49481 | 12S | PF82 | Taquari-Antas | Prata | Muitos Capões, RS | 28°21'53"S 51°17'53”W | MT803137 |
| *P. hystrix* | MCP 22799 | MORPH | PH249 | Taquari-Antas | Prata | Muitos Capões, RS | 28°21'51"S 51°17'53”W | - |
| *P. hystrix* | MCP 22799 | MORPH | PH250 | Taquari-Antas | Prata | Muitos Capões, RS | 28°21'51"S 51°17'53”W | - |
| *P. hystrix* | MCP 49470 | *coI* | PF81 | Taquari-Antas | Prata | Lagoa Vermelha, RS | 28°16'26"S 51°28'07”W | MT802348 |
| *P. hystrix* | MCP 49470 | *cytb* | PF81 | Taquari-Antas | Prata | Lagoa Vermelha, RS | 28°16'26"S 51°28'07”W | MT809658 |
| *P. hystrix* | MCP 49470 | 16S | PF81 | Taquari-Antas | Prata | Lagoa Vermelha, RS | 28°16'26"S 51°28'07”W | MT799925 |
| *P. hystrix* | MCP 49470 | 12S | PF81 | Taquari-Antas | Prata | Lagoa Vermelha, RS | 28°16'26"S 51°28'07”W | MT803136 |
| *P. hystrix* | MCP 22788 | MORPH | PH248 | Taquari-Antas | Prata | Lagoa Vermelha, RS | 28°17'36"S 51°24'42”W | - |
| *P. hystrix* | MCP 49497 | MORPH | PH240 | Taquari-Antas | Prata | Lagoa Vermelha, RS | 28°24'19"S 51°29'25”W | - |
| *P. hystrix* | MCP 49497 | MORPH | PH243 | Taquari-Antas | Prata | Lagoa Vermelha, RS | 28°24'19"S 51°29'25”W | - |
| *P. hystrix* | MCP 49497 | MORPH | PH244 | Taquari-Antas | Prata | Lagoa Vermelha, RS | 28°24'19"S 51°29'25”W | - |
| *P. hystrix* | MCP 35054 | MORPH | PH236 | Taquari-Antas | Prata | Lagoa Vermelha, RS | 28°24'19"S 51°29'25”W | - |
| *P. hystrix* | MCP 35054 | MORPH | PH237 | Taquari-Antas | Prata | Lagoa Vermelha, RS | 28°24'19"S 51°29'25”W | - |
| *P. hystrix* | MCP 35054 | MORPH | PH241 | Taquari-Antas | Prata | Lagoa Vermelha, RS | 28°24'19"S 51°29'25”W | - |
| *P. hystrix* | MCP 35054 | MORPH | PH242 | Taquari-Antas | Prata | Lagoa Vermelha, RS | 28°24'19"S 51°29'25”W | - |
| *P. hystrix* | MCP 49478 | MORPH | PH246 | Taquari-Antas | Prata | Turvo, RS | 28°25'52"S 51°29'39”W | - |
| *P. hystrix* | MCP 49478 | *cytb* | PF80 | Taquari-Antas | Prata | Turvo, RS | 28°25'52"S 51°29'39”W | MT809657 |
| *P. hystrix* | MCP 49478 | 16S | PF80 | Taquari-Antas | Prata | Turvo, RS | 28°25'52"S 51°29'39”W | MT799924 |
| *P. hystrix* | MCP 49478 | 12S | PF80 | Taquari-Antas | Prata | Turvo, RS | 28°25'52"S 51°29'39”W | MT803135 |
| *P. hystrix* | MCP 35053 | MORPH | PH245 | Taquari-Antas | Prata | André da Rocha, RS | 28°39'35"S 51°37'05”W | - |
| *P. hystrix* | MCP 50356 | MORPH | PH234 | Taquari-Antas | Prata | André da Rocha, RS | 28°39'34"S 51°37'04”W | - |
| *P. hystrix* | MCP 50356 | MORPH | PH235 | Taquari-Antas | Prata | André da Rocha, RS | 28°39'34"S 51°37'04”W | - |
| *P. hystrix* | MCP 50356 | *coI* | PF55 | Taquari-Antas | Prata | André da Rocha, RS | 28°39'34"S 51°37'04”W | MT802332 |
| *P. hystrix* | MCP 50356 | *cytb* | PF55 | Taquari-Antas | Prata | André da Rocha, RS | 28°39'34"S 51°37'04”W | MT809640 |
| *P. hystrix* | MCP 50356 | 16S | PF55 | Taquari-Antas | Prata | André da Rocha, RS | 28°39'34"S 51°37'04”W | MT799907 |
| *P. hystrix* | MCP 50356 | 12S | PF55 | Taquari-Antas | Prata | André da Rocha, RS | 28°39'34"S 51°37'04”W | MT803118 |
| *P. hystrix* | MCP 49485 | MORPH | PH238 | Taquari-Antas | Prata | Vila Flores, RS | 28°52'48"S 51°35'19”W | - |
| *P. hystrix* | MCP 49485 | MORPH | PH239 | Taquari-Antas | Prata | Vila Flores, RS | 28°52'48"S 51°35'19”W | - |
| *P. hystrix* | MCP 49485 | *coI* | PF59 | Taquari-Antas | Prata | Vila Flores, RS | 28°52'48"S 51°35'19”W | MT802333 |
| *P. hystrix* | MCP 49485 | *cytb* | PF59 | Taquari-Antas | Prata | Vila Flores, RS | 28°52'48"S 51°35'19”W | MT809641 |
| *P. hystrix* | MCP 49485 | 16S | PF59 | Taquari-Antas | Prata | Vila Flores, RS | 28°52'48"S 51°35'19”W | MT799908 |
| *P. hystrix* | MCP 49485 | 12S | PF59 | Taquari-Antas | Prata | Vila Flores, RS | 28°52'48"S 51°35'19”W | MT803119 |
| *P. hystrix* | MCP 22348 | MORPH | PH122 | Uruguay | Pelotas | Bom Jardim da Serra, SC | 28°13'45"S 49°36'03”W | - |
| *P. hystrix* | MCP 22350 | MORPH | PH121 | Uruguay | Pelotas | Bom Jardim da Serra, SC | 28°18'26"S 49°37'02”W | - |
| *P. hystrix* | MCP 22347 | MORPH | PH124 | Uruguay | Pelotas | Bom Jardim da Serra, SC | 28°20'15"S 49°37'49”W | - |
| *P. hystrix* | MCP 22345 | MORPH | PH123 | Uruguay | Pelotas | Bom Jardim da Serra, SC | 28°24'21"S 49°38'26”W | - |
| *P. hystrix* | UFRGS 8152 | MORPH | PH113 | Uruguay | Pelotas | Bom Jardim da Serra, SC | 28°24'20"S 49°38'26”W | - |
| *P. hystrix* | UFRGS 7134 | MORPH | PH112 | Uruguay | Pelotas | Bom Jardim da Serra, SC | 28°28'46"S 49°43'20”W | - |
| *P. hystrix* | MCP 22340 | MORPH | PH125 | Uruguay | Pelotas | Urubici, SC | 28°08'14"S 49°38'55”W | - |
| *P. hystrix* | UFRGS 9552 | MORPH | PH118 | Uruguay | Pelotas | São José dos Ausentes, RS | 28°42'24"S 49°55'41”W | - |
| *P. hystrix* | UFRGS 9552 | MORPH | PH119 | Uruguay | Pelotas | São José dos Ausentes, RS | 28°42'24"S 49°55'41”W | - |
| *P. hystrix* | UFRGS 9553 | MORPH | PH120 | Uruguay | Pelotas | São José dos Ausentes, RS | 28°42'24"S 49°55'41”W | - |
| *P. hystrix* | UFRGS 9553 | MORPH | PH126 | Uruguay | Pelotas | São José dos Ausentes, RS | 28°42'24"S 49°55'41”W | - |
| *P. hystrix* | UFRGS 9554 | MORPH | PH111 | Uruguay | Pelotas | São José dos Ausentes, RS | 28°42'24"S 49°55'41”W | - |
| *P. hystrix* | UFRGS 21876 | MORPH | PH110 | Uruguay | Pelotas | São José dos Ausentes, RS | 28°40'16"S 49°57'56”W | - |
| *P. hystrix* | UFRGS 21907 | *cytb* | PF39 | Uruguay | Pelotas | São José dos Ausentes, RS | 28°40'26"S 49°58'00”W | MT809633 |
| *P. hystrix* | UFRGS 21907 | 16S | PF39 | Uruguay | Pelotas | São José dos Ausentes, RS | 28°40'26"S 49°58'00”W | MT799900 |
| *P. hystrix* | UFRGS 21907 | 12S | PF39 | Uruguay | Pelotas | São José dos Ausentes, RS | 28°40'26"S 49°58'00”W | MT803111 |
| *P. hystrix* | UFRGS 9551 | MORPH | PH109 | Uruguay | Pelotas | São José dos Ausentes, RS | 28°35'53"S 49°58'07”W | - |
| *P. hystrix* | UFRGS 9541 | MORPH | PH115 | Uruguay | Pelotas | São José dos Ausentes, RS | 28°36'07"S 49°58'57”W | - |
| *P. hystrix* | UFRGS 9541 | MORPH | PH116 | Uruguay | Pelotas | São José dos Ausentes, RS | 28°36'07"S 49°58'57”W | - |
| *P. hystrix* | UFRGS 9541 | MORPH | PH117 | Uruguay | Pelotas | São José dos Ausentes, RS | 28°36'07"S 49°58'57”W | - |
| *P. hystrix* | MCP 49988 | MORPH | PH108 | Uruguay | Pelotas | São José dos Ausentes, RS | 28°41'40"S 50°07'49”W | - |
| *P. hystrix* | MCP 49988 | *coI* | PF31 | Uruguay | Pelotas | São José dos Ausentes, RS | 28°41'40"S 50°07'49”W | MT802325 |
| *P. hystrix* | MCP 49988 | *cytb* | PF31 | Uruguay | Pelotas | São José dos Ausentes, RS | 28°41'40"S 50°07'49”W | MT809629 |
| *P. hystrix* | MCP 49988 | 16S | PF31 | Uruguay | Pelotas | São José dos Ausentes, RS | 28°41'40"S 50°07'49”W | MT799896 |
| *P. hystrix* | MCP 49988 | 12S | PF31 | Uruguay | Pelotas | São José dos Ausentes, RS | 28°41'40"S 50°07'49”W | MT803107 |
| *P. hystrix* | MCP 50164 | *coI* | PF33 | Uruguay | Pelotas | Silveira, RS | 28°36'22"S 49°56'06”W | MT802327 |
| *P. hystrix* | MCP 50164 | *cytb* | PF33 | Uruguay | Pelotas | Silveira, RS | 28°36'22"S 49°56'06”W | MT809631 |
| *P. hystrix* | MCP 50164 | 16S | PF33 | Uruguay | Pelotas | Silveira, RS | 28°36'22"S 49°56'06”W | MT799898 |
| *P. hystrix* | MCP 50164 | 12S | PF33 | Uruguay | Pelotas | Silveira, RS | 28°36'22"S 49°56'06”W | MT803109 |
| *P. hystrix* | MCP 49994 | *coI* | PF32 | Uruguay | Pelotas | Silveira, RS | 28°38'08"S 50°02'54”W | MT802326 |
| *P. hystrix* | MCP 49994 | *cytb* | PF32 | Uruguay | Pelotas | Silveira, RS | 28°38'08"S 50°02'54”W | MT809630 |
| *P. hystrix* | MCP 49994 | 16S | PF32 | Uruguay | Pelotas | Silveira, RS | 28°38'08"S 50°02'54”W | MT799897 |
| *P. hystrix* | MCP 49994 | 12S | PF32 | Uruguay | Pelotas | Silveira, RS | 28°38'08"S 50°02'54”W | MT803108 |
| *P. hystrix* | UFRGS 7163 | MORPH | PH114 | Uruguay | Pelotas | São Joaquim, SC | 28°15'03"S 49°58'16”W | - |
| *P. hystrix* | MCP 53198 | MORPH | PH127 | Uruguay | Pelotas | Bom Jesus, RS | 28°32'57"S 50°11'24”W | - |
| *P. hystrix* | MCP 53198 | MORPH | PH128 | Uruguay | Pelotas | Bom Jesus, RS | 28°32'57"S 50°11'24”W | - |
| *P. hystrix* | MCP 50023 | MORPH | PH104 | Uruguay | Pelotas | Bom Jesus, RS | 28°37'29"S 50°14'20”W | - |
| *P. hystrix* | MCP 50023 | MORPH | PH105 | Uruguay | Pelotas | Bom Jesus, RS | 28°37'29"S 50°14'20”W | - |
| *P. hystrix* | MCP 50023 | MORPH | PH106 | Uruguay | Pelotas | Bom Jesus, RS | 28°37'29"S 50°14'20”W | - |
| *P. hystrix* | MCP 50023 | *coI* | PF34 | Uruguay | Pelotas | Bom Jesus, RS | 28°37'29"S 50°14'20”W | MT802328 |
| *P. hystrix* | MCP 50023 | *cytb* | PF34 | Uruguay | Pelotas | Bom Jesus, RS | 28°37'29"S 50°14'20”W | MT809632 |
| *P. hystrix* | MCP 50023 | 16S | PF34 | Uruguay | Pelotas | Bom Jesus, RS | 28°37'29"S 50°14'20”W | MT799899 |
| *P. hystrix* | MCP 50023 | 12S | PF34 | Uruguay | Pelotas | Bom Jesus, RS | 28°37'29"S 50°14'20”W | MT803110 |
| *P. hystrix* | MCP 13764 | MORPH | PH107 | Uruguay | Pelotas | Bom Jesus, RS | ≈ 28°36'S 50°24'W | - |
| *P. hystrix* | MCP 53241 | MORPH | PH277 | Uruguay | Canoas | Ponte Alta, SC | 27°29'13"S 50°22'37”W | - |
| *P. hystrix* | MCP 53241 | MORPH | PH260 | Uruguay | Canoas | Ponte Alta, SC | 27°29'13"S 50°22'37”W | - |
| *P. hystrix* | MCP 53241 | MORPH | PH261 | Uruguay | Canoas | Ponte Alta, SC | 27°29'13"S 50°22'37”W | - |
| *P. hystrix* | MCP 53241 | MORPH | PH262 | Uruguay | Canoas | Ponte Alta, SC | 27°29'13"S 50°22'37”W | - |
| *P. hystrix* | MCP 53241 | MORPH | PH263 | Uruguay | Canoas | Ponte Alta, SC | 27°29'13"S 50°22'37”W | - |
| *P. hystrix* | MCP 53241 | MORPH | PH264 | Uruguay | Canoas | Ponte Alta, SC | 27°29'13"S 50°22'37”W | - |
| *P. hystrix* | MCP 53241 | MORPH | PH265 | Uruguay | Canoas | Ponte Alta, SC | 27°29'13"S 50°22'37”W | - |
| *P. hystrix* | MCP 53241 | MORPH | PH266 | Uruguay | Canoas | Ponte Alta, SC | 27°29'13"S 50°22'37”W | - |
| *P. hystrix* | MCP 53241 | MORPH | PH267 | Uruguay | Canoas | Ponte Alta, SC | 27°29'13"S 50°22'37”W | - |
| *P. hystrix* | MCP 53241 | MORPH | PH268 | Uruguay | Canoas | Ponte Alta, SC | 27°29'13"S 50°22'37”W | - |
| *P. hystrix* | MCP 53241 | MORPH | PH269 | Uruguay | Canoas | Ponte Alta, SC | 27°29'13"S 50°22'37”W | - |
| *P. hystrix* | MCP 53241 | MORPH | PH270 | Uruguay | Canoas | Ponte Alta, SC | 27°29'13"S 50°22'37”W | - |
| *P. hystrix* | MCP 53241 | MORPH | PH271 | Uruguay | Canoas | Ponte Alta, SC | 27°29'13"S 50°22'37”W | - |
| *P. hystrix* | MCP 53241 | MORPH | PH272 | Uruguay | Canoas | Ponte Alta, SC | 27°29'13"S 50°22'37”W | - |
| *P. hystrix* | MCP 53241 | MORPH | PH273 | Uruguay | Canoas | Ponte Alta, SC | 27°29'13"S 50°22'37”W | - |
| *P. hystrix* | MCP 53241 | MORPH | PH274 | Uruguay | Canoas | Ponte Alta, SC | 27°29'13"S 50°22'37”W | - |
| *P. hystrix* | MCP 53241 | MORPH | PH275 | Uruguay | Canoas | Ponte Alta, SC | 27°29'13"S 50°22'37”W | - |
| *P. hystrix* | MCP 53241 | MORPH | PH276 | Uruguay | Canoas | Ponte Alta, SC | 27°29'13"S 50°22'37”W | - |
| *P. hystrix* | MCP 53241 | MORPH | PH278 | Uruguay | Canoas | Ponte Alta, SC | 27°29'13"S 50°22'37”W | - |
| *P. hystrix* | MCP 53241 | MORPH | PH279 | Uruguay | Canoas | Ponte Alta, SC | 27°29'13"S 50°22'37”W | - |
| *P. hystrix* | MCP 53241 | MORPH | PH280 | Uruguay | Canoas | Ponte Alta, SC | 27°29'13"S 50°22'37”W | - |
| *P. hystrix* | MCP 53241 | MORPH | PH281 | Uruguay | Canoas | Ponte Alta, SC | 27°29'13"S 50°22'37”W | - |
| *P. hystrix* | MCP 53241 | MORPH | PH282 | Uruguay | Canoas | Ponte Alta, SC | 27°29'13"S 50°22'37”W | - |
| *P. hystrix* | MCP 53241 | *coI* | PF64 | Uruguay | Canoas | Ponte Alta, SC | 27°29'13"S 50°22'37”W | MT802336 |
| *P. hystrix* | MCP 53241 | *cytb* | PF64 | Uruguay | Canoas | Ponte Alta, SC | 27°29'13"S 50°22'37”W | MT809645 |
| *P. hystrix* | MCP 53241 | 16S | PF64 | Uruguay | Canoas | Ponte Alta, SC | 27°29'13"S 50°22'37”W | MT799912 |
| *P. hystrix* | MCP 53241 | 12S | PF64 | Uruguay | Canoas | Ponte Alta, SC | 27°29'13"S 50°22'37”W | MT803123 |
| *P. hystrix* | MCP 53241 | *coI* | PF65 | Uruguay | Canoas | Ponte Alta, SC | 27°29'13"S 50°22'37”W | MT802337 |
| *P. hystrix* | MCP 53241 | *cytb* | PF65 | Uruguay | Canoas | Ponte Alta, SC | 27°29'13"S 50°22'37”W | MT809646 |
| *P. hystrix* | MCP 53241 | 16S | PF65 | Uruguay | Canoas | Ponte Alta, SC | 27°29'13"S 50°22'37”W | MT799913 |
| *P. hystrix* | MCP 53241 | 12S | PF65 | Uruguay | Canoas | Ponte Alta, SC | 27°29'13"S 50°22'37”W | MT803124 |
| *P. hystrix* | MCP 52573 | MORPH | PH259 | Uruguay | Canoas | Campo Belo do Sul, SC | 27°42'45"S 50°40'10”W | - |
| *P. hystrix* | MCP 53208 | *coI* | PF60 | Uruguay | Canoas | Campos Novos, SC | 27°23'31"S 51°09'31”W | MT802334 |
| *P. hystrix* | MCP 53208 | *cytb* | PF60 | Uruguay | Canoas | Campos Novos, SC | 27°23'31"S 51°09'31”W | MT809642 |
| *P. hystrix* | MCP 53208 | 16S | PF60 | Uruguay | Canoas | Campos Novos, SC | 27°23'31"S 51°09'31”W | MT799909 |
| *P. hystrix* | MCP 53208 | 12S | PF60 | Uruguay | Canoas | Campos Novos, SC | 27°23'31"S 51°09'31”W | MT803120 |
| *P. hystrix* | MCP 53259 | *cytb* | PF62 | Uruguay | Canoas | Campos Novos, SC | 27°32'58"S 51°26'29”W | MT809643 |
| *P. hystrix* | MCP 53259 | 16S | PF62 | Uruguay | Canoas | Campos Novos, SC | 27°32'58"S 51°26'29”W | MT799910 |
| *P. hystrix* | MCP 53259 | 12S | PF62 | Uruguay | Canoas | Campos Novos, SC | 27°32'58"S 51°26'29”W | MT803121 |
| *P. hystrix* | MCP 53259 | *coI* | PF63 | Uruguay | Canoas | Campos Novos, SC | 27°32'58"S 51°26'29”W | MT802335 |
| *P. hystrix* | MCP 53259 | *cytb* | PF63 | Uruguay | Canoas | Campos Novos, SC | 27°32'58"S 51°26'29”W | MT809644 |
| *P. hystrix* | MCP 53259 | 16S | PF63 | Uruguay | Canoas | Campos Novos, SC | 27°32'58"S 51°26'29”W | MT799911 |
| *P. hystrix* | MCP 53259 | 12S | PF63 | Uruguay | Canoas | Campos Novos, SC | 27°32'58"S 51°26'29”W | MT803122 |
| *P. hystrix* | MCP 19388 | MORPH | PH31 | Uruguay | Middle Uruguay | Tupitinga, SC | 27°33'54"S 51°23'06”W | - |
| *P. hystrix* | MCP 47753 | MORPH | PH26 | Uruguay | Middle Uruguay | Barracão, RS | 27°36'55"S 51°28'31”W | - |
| *P. hystrix* | MCP 51229 | MORPH | PH27 | Uruguay | Middle Uruguay | Paim Filho, RS | 27°40'36"S 51°44'09”W | - |
| *P. hystrix* | MCP 51229 | *coI* | PF48 | Uruguay | Middle Uruguay | Paim Filho, RS | 27°40'36"S 51°44'09”W | MT802115 |
| *P. hystrix* | MCP 51229 | *cytb* | PF48 | Uruguay | Middle Uruguay | Paim Filho, RS | 27°40'36"S 51°44'09”W | MT809636 |
| *P. hystrix* | MCP 51229 | 16S | PF48 | Uruguay | Middle Uruguay | Paim Filho, RS | 27°40'36"S 51°44'09”W | MT799903 |
| *P. hystrix* | MCP 51229 | 12S | PF48 | Uruguay | Middle Uruguay | Paim Filho, RS | 27°40'36"S 51°44'09”W | MT803114 |
| *P. hystrix* | MCP 41462 | MORPH | PH28 | Uruguay | Middle Uruguay | Machadinho, RS | 27°37'04"S 51°45'03”W | - |
| *P. hystrix* | MCP 50946 | MORPH | PH44 | Uruguay | Middle Uruguay | Machadinho, RS | 27°36'48"S 51°45'10”W | - |
| *P. hystrix* | MCP 50946 | MORPH | PH45 | Uruguay | Middle Uruguay | Machadinho, RS | 27°36'48"S 51°45'10”W | - |
| *P. hystrix* | MCP 50946 | MORPH | PH46 | Uruguay | Middle Uruguay | Machadinho, RS | 27°36'48"S 51°45'10”W | - |
| *P. hystrix* | MCP 51452 | MORPH | PH38 | Uruguay | Middle Uruguay | Maximiliano de Almeida, RS | 27°37'11"S 51°45'09”W | - |
| *P. hystrix* | MCP 51452 | MORPH | PH39 | Uruguay | Middle Uruguay | Maximiliano de Almeida, RS | 27°37'11"S 51°45'09”W | - |
| *P. hystrix* | MCP 51452 | MORPH | PH40 | Uruguay | Middle Uruguay | Maximiliano de Almeida, RS | 27°37'11"S 51°45'09”W | - |
| *P. hystrix* | MCP 51452 | MORPH | PH41 | Uruguay | Middle Uruguay | Maximiliano de Almeida, RS | 27°37'11"S 51°45'09”W | - |
| *P. hystrix* | MCP 51452 | MORPH | PH42 | Uruguay | Middle Uruguay | Maximiliano de Almeida, RS | 27°37'11"S 51°45'09”W | - |
| *P. hystrix* | MCP 51452 | MORPH | PH43 | Uruguay | Middle Uruguay | Maximiliano de Almeida, RS | 27°37'11"S 51°45'09”W | - |
| *P. hystrix* | MCP 51452 | *coI* | PF54 | Uruguay | Middle Uruguay | Maximiliano de Almeida, RS | 27°37'11"S 51°45'09”W | MT802331 |
| *P. hystrix* | MCP 51452 | *cytb* | PF54 | Uruguay | Middle Uruguay | Maximiliano de Almeida, RS | 27°37'11"S 51°45'09”W | MT809639 |
| *P. hystrix* | MCP 51452 | 16S | PF54 | Uruguay | Middle Uruguay | Maximiliano de Almeida, RS | 27°37'11"S 51°45'09”W | MT799906 |
| *P. hystrix* | MCP 51452 | 12S | PF54 | Uruguay | Middle Uruguay | Maximiliano de Almeida, RS | 27°37'11"S 51°45'09”W | MT803117 |
| *P. hystrix* | MCP 51270 | MORPH | PH30 | Uruguay | Middle Uruguay | Maximiliano de Almeida, RS | 27°36'50"S 51°45'13”W | - |
| *P. hystrix* | MCP 51270 | *coI* | PF51 | Uruguay | Middle Uruguay | Maximiliano de Almeida, RS | 27°36'50"S 51°45'13”W | MT802117 |
| *P. hystrix* | MCP 51270 | *cytb* | PF51 | Uruguay | Middle Uruguay | Maximiliano de Almeida, RS | 27°36'50"S 51°45'13”W | MT809638 |
| *P. hystrix* | MCP 51270 | 16S | PF51 | Uruguay | Middle Uruguay | Maximiliano de Almeida, RS | 27°36'50"S 51°45'13”W | MT799905 |
| *P. hystrix* | MCP 51270 | 12S | PF51 | Uruguay | Middle Uruguay | Maximiliano de Almeida, RS | 27°36'50"S 51°45'13”W | MT803116 |
| *P. hystrix* | MCP 53255 | MORPH | PH48 | Uruguay | Middle Uruguay | Maximiliano de Almeida, RS | 27°37'13"S 51°45'12”W | - |
| *P. hystrix* | MCP 53255 | MORPH | PH49 | Uruguay | Middle Uruguay | Maximiliano de Almeida, RS | 27°37'13"S 51°45'12”W | - |
| *P. hystrix* | MCP 53258 | *coI* | PF94 | Uruguay | Middle Uruguay | Barão de Cotegipe, RS | 27°39'47"S 51°29'37”W | MT802354 |
| *P. hystrix* | MCP 53258 | *cytb* | PF94 | Uruguay | Middle Uruguay | Barão de Cotegipe, RS | 27°39'47"S 51°29'37”W | MT809664 |
| *P. hystrix* | MCP 53258 | 16S | PF94 | Uruguay | Middle Uruguay | Barão de Cotegipe, RS | 27°39'47"S 51°29'37”W | MT799931 |
| *P. hystrix* | MCP 53258 | 12S | PF94 | Uruguay | Middle Uruguay | Barão de Cotegipe, RS | 27°39'47"S 51°29'37”W | MT803142 |
| *P. hystrix* | MCP 41481 | MORPH | PH34 | Uruguay | Middle Uruguay | Maximiliano de Almeida, RS | 27°38'54"S 51°45'16”W | - |
| *P. hystrix* | MCP 18800 | MORPH | PH35 | Uruguay | Middle Uruguay | Concórdia, RS | 27°38'54"S 51°45'16”W | - |
| *P. hystrix* | MCP 18800 | MORPH | PH36 | Uruguay | Middle Uruguay | Concórdia, RS | 27°38'54"S 51°45'16”W | - |
| *P. hystrix* | MCP 18800 | MORPH | PH37 | Uruguay | Middle Uruguay | Concórdia, RS | 27°38'54"S 51°45'16”W | - |
| *P. hystrix* | MCP 18799 | MORPH | PH47 | Uruguay | Middle Uruguay | Concórdia, RS | 27°19'11"S 51°55'30”W | - |
| *P. hystrix* | MCP 51286 | MORPH | PH33 | Uruguay | Middle Uruguay | Arabutã, SC | 27°09'40"S 52°08'54”W | - |
| *P. hystrix* | MCP 51286 | *coI* | PF49 | Uruguay | Middle Uruguay | Arabutã, SC | 27°09'40"S 52°08'54”W | MT802116 |
| *P. hystrix* | MCP 51286 | *cytb* | PF49 | Uruguay | Middle Uruguay | Arabutã, SC | 27°09'40"S 52°08'54”W | MT809637 |
| *P. hystrix* | MCP 51286 | 16S | PF49 | Uruguay | Middle Uruguay | Arabutã, SC | 27°09'40"S 52°08'54”W | MT799904 |
| *P. hystrix* | MCP 51286 | 12S | PF49 | Uruguay | Middle Uruguay | Arabutã, SC | 27°09'40"S 52°08'54”W | MT803115 |
| *P. hystrix* | MCP 27028 | MORPH | PH56 | Uruguay | Passo Fundo | Getúlio Vargas, RS | 27°52'60"S 52°14'00”W | - |
| *P. hystrix* | MCP 46819 | MORPH | PH54 | Uruguay | Passo Fundo | Erebango, RS | 27°48'16"S 52°20'20”W | - |
| *P. hystrix* | MCP 46772 | MORPH | PH55 | Uruguay | Passo Fundo | Barão de Cotegipe, RS | 27°37'26"S 52°23'23”W | - |
| *P. hystrix* | MCP 46772 | *cytb* | PF21A | Uruguay | Passo Fundo | Barão de Cotegipe, RS | 27°37'26"S 52°23'23”W | MT809623 |
| *P. hystrix* | MCP 46772 | 16S | PF21A | Uruguay | Passo Fundo | Barão de Cotegipe, RS | 27°37'26"S 52°23'23”W | MT799890 |
| *P. hystrix* | MCP 46772 | 12S | PF21A | Uruguay | Passo Fundo | Barão de Cotegipe, RS | 27°37'26"S 52°23'23”W | MT803101 |
| *P. hystrix* | MCP 46772 | *coI* | PF22B | Uruguay | Passo Fundo | Barão de Cotegipe, RS | 27°37'26"S 52°23'23”W | MT802320 |
| *P. hystrix* | MCP 46772 | *cytb* | PF22B | Uruguay | Passo Fundo | Barão de Cotegipe, RS | 27°37'26"S 52°23'23”W | MT809624 |
| *P. hystrix* | MCP 46772 | 16S | PF22B | Uruguay | Passo Fundo | Barão de Cotegipe, RS | 27°37'26"S 52°23'23”W | MT799891 |
| *P. hystrix* | MCP 46772 | 12S | PF22B | Uruguay | Passo Fundo | Barão de Cotegipe, RS | 27°37'26"S 52°23'23”W | MT803102 |
| *P. hystrix* | MCP 53257 | MORPH | PH64 | Uruguay | Passo Fundo | Barão de Cotegipe, RS | 27°37'26"S 52°23'23”W | - |
| *P. hystrix* | MCP 53257 | MORPH | PH65 | Uruguay | Passo Fundo | Barão de Cotegipe, RS | 27°37'26"S 52°23'23”W | - |
| *P. hystrix* | MCP 53257 | MORPH | PH66 | Uruguay | Passo Fundo | Barão de Cotegipe, RS | 27°37'26"S 52°23'23”W | - |
| *P. hystrix* | MCP 53257 | MORPH | PH67 | Uruguay | Passo Fundo | Barão de Cotegipe, RS | 27°37'26"S 52°23'23”W | - |
| *P. hystrix* | MCP 53257 | MORPH | PH68 | Uruguay | Passo Fundo | Barão de Cotegipe, RS | 27°37'26"S 52°23'23”W | - |
| *P. hystrix* | MCP 53257 | *coI* | PF75 | Uruguay | Passo Fundo | Barão de Cotegipe, RS | 27°37'26"S 52°23'23”W | MT802343 |
| *P. hystrix* | MCP 53257 | *cytb* | PF75 | Uruguay | Passo Fundo | Barão de Cotegipe, RS | 27°37'26"S 52°23'23”W | MT809652 |
| *P. hystrix* | MCP 53257 | 16S | PF75 | Uruguay | Passo Fundo | Barão de Cotegipe, RS | 27°37'26"S 52°23'23”W | MT799919 |
| *P. hystrix* | MCP 53257 | 12S | PF75 | Uruguay | Passo Fundo | Barão de Cotegipe, RS | 27°37'26"S 52°23'23”W | MT803130 |
| *P. hystrix* | MCP 53257 | *coI* | PF76 | Uruguay | Passo Fundo | Barão de Cotegipe, RS | 27°37'26"S 52°23'23”W | MT802344 |
| *P. hystrix* | MCP 53257 | *cytb* | PF76 | Uruguay | Passo Fundo | Barão de Cotegipe, RS | 27°37'26"S 52°23'23”W | MT809653 |
| *P. hystrix* | MCP 53257 | 16S | PF76 | Uruguay | Passo Fundo | Barão de Cotegipe, RS | 27°37'26"S 52°23'23”W | MT799920 |
| *P. hystrix* | MCP 53257 | 12S | PF76 | Uruguay | Passo Fundo | Barão de Cotegipe, RS | 27°37'26"S 52°23'23”W | MT803131 |
| *]P. hystrix* | MCP 47906 | MORPH | PH62 | Uruguay | Passo Fundo | Rondinha, RS | 27°55'03"S 52°50'39”W | - |
| *P. hystrix* | MCP 44272 | MORPH | PH57 | Uruguay | Passo Fundo | Nonoai, RS | 27°20'43"S 52°43'51”W | - |
| *P. hystrix* | MCP 44272 | MORPH | PH58 | Uruguay | Passo Fundo | Nonoai, RS | 27°20'43"S 52°43'51”W | - |
| *P. hystrix* | MCP 26508 | MORPH | PH53 | Uruguay | Passo Fundo | Nonoai, RS | 27°21'60"S 52°43'00”W | - |
| *P. hystrix* | MCP 43227 | MORPH | PH52 | Uruguay | Passo Fundo | Nonoai, RS | 27°20'54"S 52°43'57”W | - |
| *P. hystrix* | MCP 44271 | MORPH | PH60 | Uruguay | Passo Fundo | Nonoai, RS | 27°20'43"S 52°43'51”W | - |
| *P. hystrix* | MCP 44271 | MORPH | PH61 | Uruguay | Passo Fundo | Nonoai, RS | 27°20'43"S 52°43'51”W | - |
| *P. hystrix* | MCP 53254 | MORPH | PH63 | Uruguay | Passo Fundo | Nonoai, RS | 27°20'04"S 52°44'09"W | - |
| *P. hystrix* | MCP 53254 | *coI* | PF74 | Uruguay | Passo Fundo | Nonoai, RS | 27°20'04"S 52°44'09"W | MT802342 |
| *P. hystrix* | MCP 53254 | *cytb* | PF74 | Uruguay | Passo Fundo | Nonoai, RS | 27°20'04"S 52°44'09"W | MT809651 |
| *P. hystrix* | MCP 53254 | 16S | PF74 | Uruguay | Passo Fundo | Nonoai, RS | 27°20'04"S 52°44'09"W | MT799918 |
| *P. hystrix* | MCP 53254 | 12S | PF74 | Uruguay | Passo Fundo | Nonoai, RS | 27°20'04"S 52°44'09"W | MT803129 |
| *P. hystrix* | MCP 40058 | MORPH | PH81 | Uruguay | Chapecó | Aberlado Luz, SC | 26°33'03"S 52°19'10”W | - |
| *P. hystrix* | MCP 40058 | MORPH | PH82 | Uruguay | Chapecó | Aberlado Luz, SC | 26°33'03"S 52°19'10”W | - |
| *P. hystrix* | MCP 40058 | MORPH | PH83 | Uruguay | Chapecó | Aberlado Luz, SC | 26°33'03"S 52°19'10”W | - |
| *P. hystrix* | MCP 40058 | MORPH | PH98 | Uruguay | Chapecó | Aberlado Luz, SC | 26°33'03"S 52°19'10”W | - |
| *P. hystrix* | MCP 48283 | *coI* | PF28A | Uruguay | Chapecó | Aberlado Luz, SC | 26°33'05"S 52°19'10”W | MT802322 |
| *P. hystrix* | MCP 48283 | *cytb* | PF28A | Uruguay | Chapecó | Aberlado Luz, SC | 26°33'05"S 52°19'10”W | MT809626 |
| *P. hystrix* | MCP 48283 | 16S | PF28A | Uruguay | Chapecó | Aberlado Luz, SC | 26°33'05"S 52°19'10”W | MT799893 |
| *P. hystrix* | MCP 48283 | 12S | PF28A | Uruguay | Chapecó | Aberlado Luz, SC | 26°33'05"S 52°19'10”W | MT803104 |
| *P. hystrix* | MCP 48283 | *coI* | PF29B | Uruguay | Chapecó | Aberlado Luz, SC | 26°33'05"S 52°19'10”W | MT802323 |
| *P. hystrix* | MCP 48283 | *cytb* | PF29B | Uruguay | Chapecó | Aberlado Luz, SC | 26°33'05"S 52°19'10”W | MT809627 |
| *P. hystrix* | MCP 48283 | 16S | PF29B | Uruguay | Chapecó | Aberlado Luz, SC | 26°33'05"S 52°19'10”W | MT799894 |
| *P. hystrix* | MCP 48283 | 12S | PF29B | Uruguay | Chapecó | Aberlado Luz, SC | 26°33'05"S 52°19'10”W | MT803105 |
| *P. hystrix* | MCP 48283 | *coI* | PF30C | Uruguay | Chapecó | Aberlado Luz, SC | 26°33'05"S 52°19'10”W | MT802324 |
| *P. hystrix* | MCP 48283 | *cytb* | PF30C | Uruguay | Chapecó | Aberlado Luz, SC | 26°33'05"S 52°19'10”W | MT809628 |
| *P. hystrix* | MCP 48283 | 16S | PF30C | Uruguay | Chapecó | Aberlado Luz, SC | 26°33'05"S 52°19'10”W | MT799895 |
| *P. hystrix* | MCP 48283 | 12S | PF30C | Uruguay | Chapecó | Aberlado Luz, SC | 26°33'05"S 52°19'10”W | MT803106 |
| *P. hystrix* | MCP 40150 | MORPH | PH79 | Uruguay | Chapecó | Aberlado Luz, SC | 26°33'35"S 52°19'41”W | - |
| *P. hystrix* | MCP 40150 | MORPH | PH80 | Uruguay | Chapecó | Aberlado Luz, SC | 26°33'35"S 52°19'41”W | - |
| *P. hystrix* | MCP 48282 | MORPH | PH97 | Uruguay | Chapecó | Chapecó, SC | 26°38'22"S 52°54'08”W | - |
| *P. hystrix* | MCP 40132 | MORPH | PH77 | Uruguay | Chapecó | Modelo, SC | 26°47'10"S 53°02'43”W | - |
| *P. hystrix* | MCP 40132 | MORPH | PH78 | Uruguay | Chapecó | Modelo, SC | 26°47'10"S 53°02'43”W | - |
| *P. hystrix* | MCP 40132 | *coI* | PF27 | Uruguay | Chapecó | Modelo, SC | 26°47'10"S 53°02'43”W | MT802321 |
| *P. hystrix* | MCP 40132 | *cytb* | PF27 | Uruguay | Chapecó | Modelo, SC | 26°47'10"S 53°02'43”W | MT809625 |
| *P. hystrix* | MCP 40132 | 16S | PF27 | Uruguay | Chapecó | Modelo, SC | 26°47'10"S 53°02'43”W | MT799892 |
| *P. hystrix* | MCP 40132 | 12S | PF27 | Uruguay | Chapecó | Modelo, SC | 26°47'10"S 53°02'43”W | MT803103 |
| *P. hystrix* | MCP 53263 | MORPH | PH92 | Uruguay | Chapecó | Modelo, SC | 26°47'11"S 53°02'58”W | - |
| *P. hystrix* | MCP 53263 | MORPH | PH93 | Uruguay | Chapecó | Modelo, SC | 26°47'11"S 53°02'58”W | - |
| *P. hystrix* | MCP 53263 | MORPH | PH94 | Uruguay | Chapecó | Modelo, SC | 26°47'11"S 53°02'58”W | - |
| *P. hystrix* | MCP 53263 | MORPH | PH95 | Uruguay | Chapecó | Modelo, SC | 26°47'11"S 53°02'58”W | - |
| *P. hystrix* | MCP 53263 | MORPH | PH96 | Uruguay | Chapecó | Modelo, SC | 26°47'11"S 53°02'58”W | - |
| *P. hystrix* | MCP 53263 | *coI* | PF77 | Uruguay | Chapecó | Modelo, SC | 26°47'11"S 53°02'58”W | MT802345 |
| *P. hystrix* | MCP 53263 | *cytb* | PF77 | Uruguay | Chapecó | Modelo, SC | 26°47'11"S 53°02'58”W | MT809654 |
| *P. hystrix* | MCP 53263 | 16S | PF77 | Uruguay | Chapecó | Modelo, SC | 26°47'11"S 53°02'58”W | MT799921 |
| *P. hystrix* | MCP 53263 | 12S | PF77 | Uruguay | Chapecó | Modelo, SC | 26°47'11"S 53°02'58”W | MT803132 |
| *P. hystrix* | MCP 21114 | MORPH | PH207 | Uruguay | Ijuí | Condor, RS | ≈ 28°12'S 53°31'W | - |
| *P. hystrix* | MCP 21115 | MORPH | PH203 | Uruguay | Ijuí | Condor, RS | ≈ 28°13'S 53°33'W | - |
| *P. hystrix* | MCP 21191 | MORPH | PH209 | Uruguay | Ijuí | Condor, RS | ≈ 28°11'S 53°27'W | - |
| *P. hystrix* | MCP 21191 | MORPH | PH210 | Uruguay | Ijuí | Condor, RS | ≈ 28°11'S 53°27'W | - |
| *P. hystrix* | MCP 18741 | MORPH | PH211 | Uruguay | Ijuí | Condor, RS | ≈ 28°11'S 53°27'W | - |
| *P. hystrix* | MCP 53273 | *coI* | PF73 | Uruguay | Ijuí | Pejuçara, RS | 28°19'37"S 53°39'19"W | MT802341 |
| *P. hystrix* | MCP 53273 | *cytb* | PF73 | Uruguay | Ijuí | Pejuçara, RS | 28°19'37"S 53°39'19"W | MT809650 |
| *P. hystrix* | MCP 53273 | 16S | PF73 | Uruguay | Ijuí | Pejuçara, RS | 28°19'37"S 53°39'19"W | MT799917 |
| *P. hystrix* | MCP 53273 | 12S | PF73 | Uruguay | Ijuí | Pejuçara, RS | 28°19'37"S 53°39'19"W | MT803128 |
| *P. hystrix* | MCP 53274 | MORPH | PH224 | Uruguay | Ijuí | Condor, RS | 28°12'31"S 28°12'31”W | - |
| *P. hystrix* | MCP 53274 | MORPH | PH225 | Uruguay | Ijuí | Condor, RS | 28°12'31"S 28°12'31”W | - |
| *P. hystrix* | MCP 53274 | MORPH | PH226 | Uruguay | Ijuí | Condor, RS | 28°12'31"S 28°12'31”W | - |
| *P. hystrix* | MCP 53274 | MORPH | PH227 | Uruguay | Ijuí | Condor, RS | 28°12'31"S 28°12'31”W | - |
| *P. hystrix* | MCP 53274 | MORPH | PH228 | Uruguay | Ijuí | Condor, RS | 28°12'31"S 28°12'31”W | - |
| *P. hystrix* | MCP 49204 | MORPH | PH208 | Uruguay | Ijuí | Panambi, RS | 28°14'35"S 53°33'12”W | - |
| *P. hystrix* | MCP 48639 | MORPH | PH218 | Uruguay | Ijuí | Panambi, RS | 28°15'03"S 53°33'49”W | - |
| *P. hystrix* | UFRGS 11704 | MORPH | PH219 | Uruguay | Ijuí | Panambi, RS | 28°13'19"S 53°34'05”W | - |
| *P. hystrix* | MCP 50927 | *cytb* | PF13 | Uruguay | Ijuí | Ijuí, RS | 28°22'10"S 53°52'43”W | MT809620 |
| *P. hystrix* | MCP 50927 | 16S | PF13 | Uruguay | Ijuí | Ijuí, RS | 28°22'10"S 53°52'43”W | MT799887 |
| *P. hystrix* | MCP 50927 | 12S | PF13 | Uruguay | Ijuí | Ijuí, RS | 28°22'10"S 53°52'43”W | MT803098 |
| *P. hystrix* | MCP 50127 | *coI* | PF14A | Uruguay | Ijuí | Ijuí, RS | 28°22'09"N 53°52'43”W | MT802318 |
| *P. hystrix* | MCP 50127 | *cytb* | PF14A | Uruguay | Ijuí | Ijuí, RS | 28°22'09"N 53°52'43”W | MT809621 |
| *P. hystrix* | MCP 50127 | 16S | PF14A | Uruguay | Ijuí | Ijuí, RS | 28°22'09"N 53°52'43”W | MT799888 |
| *P. hystrix* | MCP 50127 | 12S | PF14A | Uruguay | Ijuí | Ijuí, RS | 28°22'09"N 53°52'43”W | MT803099 |
| *P. hystrix* | MCP 50127 | *coI* | PF16C | Uruguay | Ijuí | Ijuí, RS | 28°22'09"N 53°52'43”W | MT802319 |
| *P. hystrix* | MCP 50127 | *cytb* | PF16C | Uruguay | Ijuí | Ijuí, RS | 28°22'09"N 53°52'43”W | MT809622 |
| *P. hystrix* | MCP 50127 | 16S | PF16C | Uruguay | Ijuí | Ijuí, RS | 28°22'09"N 53°52'43”W | MT799889 |
| *P. hystrix* | MCP 50127 | 12S | PF16C | Uruguay | Ijuí | Ijuí, RS | 28°22'09"N 53°52'43”W | MT803100 |
| *P. hystrix* | MCP 32562 | MORPH | PH223 | Uruguay | Ijuí | Ijuí, RS | 28°25'00"S 53°47'00”W | - |
| *P. hystrix* | MCP 41707 | MORPH | PH204 | Uruguay | Ijuí | Bozano, RS | 28°24'00"S 53°48'00”W | - |
| *P. hystrix* | MCP 41707 | MORPH | PH205 | Uruguay | Ijuí | Bozano, RS | 28°24'00"S 53°48'00”W | - |
| *P. hystrix* | MCP 41707 | MORPH | PH206 | Uruguay | Ijuí | Bozano, RS | 28°24'00"S 53°48'00”W | - |
| *P. hystrix* | MCP 41698 | MORPH | PH215 | Uruguay | Ijuí | Bozano, RS | ≈ 28°24'S 53°48'W | - |
| *P. hystrix* | MCP 41698 | MORPH | PH 216 | Uruguay | Ijuí | Bozano, RS | ≈ 28°24'S 53°48'W | - |
| *P. hystrix* | MCP 41698 | MORPH | PH 217 | Uruguay | Ijuí | Bozano, RS | ≈ 28°24'S 53°48'W | - |
| *P. hystrix* | MCP 44995 | MORPH | PH222 | Uruguay | Ijuí | Ijuí, RS | 28°28'38"S 53°56'02”W | - |
| *P. hystrix* | MCP 44993 | MORPH | PH212 | Uruguay | Ijuí | Ijuí, RS | 28°28'12"S 53°56'52”W | - |
| *P. hystrix* | MCP 44993 | MORPH | PH213 | Uruguay | Ijuí | Ijuí, RS | 28°28'12"S 53°56'52”W | - |
| *P. hystrix* | UFRGS 6034 | MORPH | PH220 | Uruguay | Ijuí | Ijuí, RS | 28°19'00"S 53°58'00”W | - |
| *P. hystrix* | UFRGS 6034 | MORPH | PH221 | Uruguay | Ijuí | Ijuí, RS | 28°19'00"S 53°58'00”W | - |
| *P. hystrix* | MCP 16792 | MORPH | PH214 | Uruguay | Ijuí | Entre Ijuís, RS | ≈ 28°27'S 54°22'W | - |
| *P. azygolechis* | MCP 41909 | *coI* | PF103 | Coastal | Araraquara | Guaratuba, PR | 25°57'56"S 48°49'43”W | MT802357 |
| *P. azygolechis* | MCP 41909 | *cytb* | PF103 | Coastal | Araraquara | Guaratuba, PR | 25°57'56"S 48°49'43”W | MT809667 |
| *P. azygolechis* | MCP 41909 | 16S | PF103 | Coastal | Araraquara | Guaratuba, PR | 25°57'56"S 48°49'43”W | MT799934 |
| *P. azygolechis* | MCP 41909 | 12S | PF103 | Coastal | Araraquara | Guaratuba, PR | 25°57'56"S 48°49'43”W | MT803145 |
| *P. steindachneri* | MCP 41289 | *coI* | PF100 | Coastal | Cubatão | Joinville, SC | 26°10'31"S 48°57'10”W | MT802355 |
| *P. steindachneri* | MCP 41289 | *cytb* | PF100 | Coastal | Cubatão | Joinville, SC | 26°10'31"S 48°57'10”W | MT809665 |
| *P. steindachneri* | MCP 41289 | 16S | PF100 | Coastal | Cubatão | Joinville, SC | 26°10'31"S 48°57'10”W | MT799932 |
| *P. steindachneri* | MCP 41289 | 12S | PF100 | Coastal | Cubatão | Joinville, SC | 26°10'31"S 48°57'10”W | MT803143 |
| *P. vestigipinnis* | UFRGS 14619 | *coI* | PF125 | Uruguay | Canoas | Painel, SC | 27°54'34"S 50°05'15”W | MT802359 |
| *P. vestigipinnis* | UFRGS 14619 | *cytb* | PF125 | Uruguay | Canoas | Painel, SC | 27°54'34"S 50°05'15”W | MT809669 |
| *P. vestigipinnis* | UFRGS 14619 | 16S | PF125 | Uruguay | Canoas | Painel, SC | 27°54'34"S 50°05'15”W | MT799936 |
| *P. vestigipinnis* | UFRGS 14619 | 12S | PF125 | Uruguay | Canoas | Painel, SC | 27°54'34"S 50°05'15”W | MT803147 |
| *P. vestigipinnis* | MCP 43034 | *coI* | PF102 | Uruguay | Canoas | Painel, SC | 27°54'12"S 50°04'19”W | MT802356 |
| *P. vestigipinnis* | MCP 43034 | *cytb* | PF102 | Uruguay | Canoas | Painel, SC | 27°54'12"S 50°04'19”W | MT809666 |
| *P. vestigipinnis* | MCP 43034 | 16S | PF102 | Uruguay | Canoas | Painel, SC | 27°54'12"S 50°04'19”W | MT799933 |
| *P. vestigipinnis* | MCP 43034 | 12S | PF102 | Uruguay | Canoas | Painel, SC | 27°54'12"S 50°04'19”W | MT803144 |
| *P. vestigipinnis* | MCP 43034 | *coI* | PF113 | Uruguay | Canoas | Painel, SC | 27°54'12"S 50°04'19”W | MT802358 |
| *P. vestigipinnis* | MCP 43034 | *cytb* | PF113 | Uruguay | Canoas | Painel, SC | 27°54'12"S 50°04'19”W | MT809668 |
| *P. vestigipinnis* | MCP 43034 | 16S | PF113 | Uruguay | Canoas | Painel, SC | 27°54'12"S 50°04'19”W | MT799935 |
| *P. vestigipinnis* | MCP 43034 | 12S | PF113 | Uruguay | Canoas | Painel, SC | 27°54'12"S 50°04'19”W | MT803146 |
