# Supplementary material for: Iterative taxonomic study of Pareiorhaphis hystrix (Siluriformes, Loricariidae) suggests a single, yet phenotypically variable, species in south Brazil
Source: PLoS One. 2020 Sep 3;15(9):e0237160. doi: 10.1371/journal.pone.0237160 (PMC7470336; doi:10.1371/journal.pone.0237160)
Supplement: Attachment — Submitted filename: Response to reviewer.docx [file pone.0237160.s002.docx]

Response to reviewers

We thanks both reviewers and the editor for the careful review and suggestions that improved the manuscript. Below we present in blue how we treated each suggestion.

We hope that manuscript in now acceptable for publication in PlosOne.

Best, Roberto

-----------------------------------------------------------------------

1. Please ensure that your manuscript meets PLOS ONE's style requirements, including those for file naming.

We´ve made our best to ensure that the manuscript meets PlosOne style.

2. In your Methods section, please provide additional information regarding the permits you obtained for the work. Please ensure you have included the full name of the authority that approved the field site access and, if no permits were required, a brief statement explaining why.

Information on collecting permit added.

3. We note that you have stated that you will provide repository information for your data at acceptance. Should your manuscript be accepted for publication, we will hold it until you provide the relevant accession numbers or DOIs necessary to access your data. If you wish to make changes to your Data Availability statement, please describe these changes in your cover letter and we will update your Data Availability statement to reflect the information you provide.

All DNA sequences are being deposited in Genbank and their accession numbers will be included in the manuscript upon acceptance.

Reviewers' comments:

Reviewer #1: This is an important contribution to the understanding of Neotropical Fishes. The study has up-to-date methods that were implemented comprehensively to test the hypotheses proposed in the beginning of the study. Integrative taxonomic studies, as this one, that integrate morphology and genetic data from multiple genes are the best way to test species con-specificity. The results are also very detailed described and illustrated and support the conclusion that Pareiorhaphis hystrix is composed on a single, although variable, species. My main critic suggestion is that the results of the study, especially concerning the haplotype analysis, the OUT analysis, and the coI genetic distance, support that Pareiorhaphis vestigipinnis is likely co-specific with P. hystrix. In addition, the geographic distribution of the two species are complementary, and the reduction of adipose fin is a character that is variable in other armored catfishes, and that may also be variable in this case. Authors should possibly change the paper to include the synonymization. If not, they should argue more what they defer not doing that.

*Pareiorhaphis vestigipinnis* is indeed very close to *P. hystrix* and probably derived from the latter. Despite they are very close, we believe they represent independent evolving lineages, as the former has a larger distance in the haplotype network from all *P. hystrix* populations (Fig 11). It would be expected to be more closely related to the *P. hystrix* population in the Canoas River if they were the same. Also, in the phylogeny of Fig 13 it was recovered as sister to all populations of *P. hystrix*, and not closely related on the Canoas population or any other. In addition, there are two phenotypic features that consistently differentiate the two species: the absence of an adipose fin (always present and well-developed in *P. hystrix* and the club-shaped pectoral-fin spine, simple and straight in *P. hystrix*). For these reasons, we maintain both species separate, while recognizing their close proximity. These differences are now more explicit in the discussion.

My only other suggestions: 1. Merge figures 1 and 2 into a single plate.

We prefer to keep these figures separated because if they are merged, fishes will became too small in the printed page.

2. Why Passo Fundo population does not have a H number in Fig. 11?

That was a mistake. Thanks for spotting that! It is corrected now.

Reviewer #2: The manuscript provides an important contribution to the taxonomy and evolutionary relationships of Pareiorhaphis populations from Uruguay and Jacui basins. The authors used multiple lines of evidence to investigate the identity and distribution of Pareiorhaphis hystrix. In general , they have enough result supporting their hypothesis but some issues need to be fixed before the publication. See the following items.

Major issues:
1. The authors should revise the language to improve readability.

Manuscript was sent to professional proofing company to review English grammar.

2. The authors should rewrite the introduction to ensure that the readers understand the importance of the study and the reasons why they chose the concept of iterative taxonomy instead of alternative approaches traditionally used in fish taxonomy.

The introduction was edited to more explicitly demonstrate the importance of the study to readers. In addition, we clarified why the iterative taxonomy approach was chosen as opposite to the traditional approach of using morphology or molecules alone. The iterative approach in the sense of Yeates et al (2011) was bring up to the beginning of Material and Methods, to more clearly emphasize its importance.

3. In Material and Methods, please, include PCR protocols of each gene as well as the sequencing procedures.

All PCR protocols used for each fragment and the purification/sequencing facility were included.

4. In the Results (line 194) it is mentioned that more than one pattern of development of cheek odontodes and associated lateral fleshy lob was observed in males. Figure 4 illustrates eleven patterns but there is no explanation of how to distinguish one from other.

Paragraph was modified to explain that the different patterns of odontodes development vary in in size, thickness, density, direction, and position on the sides of the head. We hope that the paragraph is more clear now on this regard.

5. The name "abdominal plates" (Line 225) sounds inappropriate for the region where these platelets occur. If the plates occur only on the pectoral girdle, consider changing the name to "pectoral plates" or "thoracic plates" updating the text where necessary.

This is a good point. However, abdominal plates (either lateral or central abdominal plates) have their use well defined in loricariid anatomical descriptions. Thoracic plates were used in the past, but felt out of use because fish have no thorax. Also, pectoral plates would remit to the same mistake… We changed the text to what we think is the most appropriate to couple with the suggestion: anterior abdominal plates.

6. PCA and LDA showed quite similar results and they are not crucial for taxonomic decisions. They just show in different ways that there are no significant differences in morphology between populations. I recommend excluding one of these analyses to avoid redundancies. Otherwise, explain clearly in Material and Methods the differences between the analyses and why the inclusion of both methods are important for the work.

While PCA indicated an overlap trend between areas, LDA detected a subtle differentiation between populations from the two hydrographic basins. We thus prefer to maintain both analyses in the article, also considering that, in fact, they are complementary and corroborate each other in suggesting a more closely phenotypical similarity between Pelotas (part of the Uruguay basin) and the Taquari basin areas. It is interesting finding such results, in view of the fact the PCA simply finds the main axes of variation of a dataset in any dimension, not discriminating whether these data belong to the same class or not. On the other hand, the LDA seeks a different approach, considering the existence of classes in the data, highlighting a linear separation of these data if it exists. We added a sentence to Material and Methods to justify the use of both analyses.

7. Review carefully the Haplotype network results. Contrary to what is explained in the text (lines 344-347), I did not see any haplotype been shared between regions in Figure 11 (maybe the phrase is in the wrong place in the text)….

A figure citation was missing. The sentence referring Fig. 11 actually says “…unique and non-shared haplotypes.” The sentence mentioning shared haplotypes between regions refers to the individual gene haplotype networks, which are depicted in figure 12. The right figure is now cited.

…. In addition, in line 366 is commented that only haplotype 17 is shared but in Figure 12 there is another haplotype being shared (H_6).

In fact, the sentence says that only Haplotype_17 is shared between the two river basins (Uruguay and Taquari). Haplotype _6 is shared between two areas of the same basin – upper and middle Antas. A few words were added to that paragraph to make it more clear.

8. The authors have several interesting results but they are poorly explored and discussed. For instance, Table 5 and Figures 11 and 12 show that Ijui and Passo Fundo are the regions with less number of haplotypes. Besides, they commented that the COI 2% threshold did not work to distinguish species, but there are other works showing that in some cases the threshold value might be larger or lower (e. g. Hypostomus) than 2%.

Indeed Ijui and Passo Fundo have few haplotypes but, depending on the genes, other areas like Pelotas or Middle Uruguay also have few haplotypes. Also, yes indeed the COI 2% threshold varies across fish groups, and this is why we commented that for this genus the species threshold seems to be lower than 2%.

Minor issues:
1. In line 64, the authors comment that Pareiorhaphis is monophyletic citing a morphology-based study. Since the authors use molecular data in their phylogenetic analysis, it is worth also informing whether molecular phylogenies for the genus are available and, in positive cases, if these works corroborate with morphology-based studies.

Unfortunately, there is no published molecular phylogenies of loricariids including more than a handful of *Pareiorhaphis* species. In these studies, the few species are monophyletic or almost so (one species clustering with the very distinct *Kronichthys* in one study); or the one with more species is highly unresolved at the nodes close to *Pareiorhaphis* and thus not conclusive. A taxonomic densely sampled phylogeny is being produced for the subfamily and this information will be available soon. A preliminary tree presented in the Second Symposium of Phylogeny of Neotropical Fishes held in Londrina (Reis et al., 2017) has a monophyletic *Pareiorhaphis* based on both molecules and morphology.

2. Inform the distribution of the genus in the Introduction and the habitats explored by its species.

Good point. Information on distribution and habitat added to the Introduction.

3. Line 67 has a missing reference. Please, check carefully all cited references.

Reference added. Other references checked throughout.

4. Line 429: "time-divergence coalescence"? Looks like it is missing some information in the Figure.

Legend of Fig. 14 corrected.
